# Tumour Size and Overall Survival in a Cohort of Patients with Unifocal Glioblastoma: A Uni- and Multivariable Prognostic Modelling and Resampling Study

**DOI:** 10.3390/cancers16071301

**Published:** 2024-03-27

**Authors:** Kavi Fatania, Russell Frood, Hitesh Mistry, Susan C. Short, James O’Connor, Andrew F. Scarsbrook, Stuart Currie

**Affiliations:** 1 Department of Radiology, Leeds Teaching Hospitals NHS Trust, Leeds General Infirmary, Leeds LS1 3EX, UKa.scarsbrook@nhs.net (A.F.S.); stuartcurrie@nhs.net (S.C.); 2 Leeds Institute of Medical Research, University of Leeds, Leeds LS2 9TJ, UK; s.c.short@leeds.ac.uk; 3 Division of Cancer Sciences, The University of Manchester, Manchester M13 9PL, UK; hitesh.mistry@manchester.ac.uk (H.M.);; 4 Department of Oncology, Leeds Teaching Hospitals NHS Trust, St James’s University Hospital, Leeds LS9 7TF, UK; 5 Department of Radiology, The Christie Hospital, Manchester M20 4BX, UK; 6 Division of Radiotherapy and Imaging, Institute of Cancer Research, London SM2 5NG, UK

**Keywords:** brain neoplasms, diagnostic imaging, glioblastoma, tumour volume, prognosis, survival analysis

## Abstract

**Simple Summary:**

Glioblastoma (GBM) is the most aggressive brain cancer in adults and there is great interest in accurate stratification of people based on their survival after surgery. These proposed stratification methods are inconsistent regarding the importance of tumour size. For 279 patients diagnosed with GBM in our institute, we calculated the diameter and volume of their tumours using their MRI scan prior to surgery and used statistical modelling to investigate (1) if tumour size was important in stratifying survival in these patients and (2) why other proposed models may or may not have shown the importance of tumour size. Our results showed that tumour diameter and volume were important for predicting the outcome of patients after we considered the extent of the surgery and that diameter was also important when all other clinical factors such as age, gender, genetic changes, and post-operative cancer treatment were taken into account.

**Abstract:**

Published models inconsistently associate glioblastoma size with overall survival (OS). This study aimed to investigate the prognostic effect of tumour size in a large cohort of patients diagnosed with GBM and interrogate how sample size and non-linear transformations may impact on the likelihood of finding a prognostic effect. In total, 279 patients with a IDH-wildtype unifocal WHO grade 4 GBM between 2014 and 2020 from a retrospective cohort were included. Uni-/multivariable association between core volume, whole volume (CV and WV), and diameter with OS was assessed with (1) Cox proportional hazard models +/− log transformation and (2) resampling with 1,000,000 repetitions and varying sample size to identify the percentage of models, which showed a significant effect of tumour size. Models adjusted for operation type and a diameter model adjusted for all clinical variables remained significant (*p* = 0.03). Multivariable resampling increased the significant effects (*p* < 0.05) of all size variables as sample size increased. Log transformation also had a large effect on the chances of a prognostic effect of WV. For models adjusted for operation type, 19.5% of WV vs. 26.3% log-WV (*n* = 50) and 69.9% WV and 89.9% log-WV (*n* = 279) were significant. In this large well-curated cohort, multivariable modelling and resampling suggest tumour volume is prognostic at larger sample sizes and with log transformation for WV.

## 1. Introduction

Glioblastoma (GBM) is the most common primary brain malignancy in adults and patients have a median overall survival (OS) of 12–15 months despite intensive oncological treatment (maximum safe surgical resection followed by adjuvant radiotherapy with concurrent temozolomide and a further six cycles of adjuvant temozolomide–the Stupp protocol) [1,2].

There has been significant interest in investigating which prognostic factors are important for patients with GBM in order to better stratify patients into different risk groups due to the increasing interest in individualised treatment strategies (‘personalised medicine’) [3]. Many such prognostic models include imaging biomarkers [4]. Magnetic resonance imaging (MRI) is routinely performed for patients with GBM throughout their treatment pathway and is therefore a popular modality for exploring prognostic imaging biomarkers. The diameter of the tumour core, which is commonly defined as the enhancing and necrotic portion of the tumour [5], is routinely evaluated in clinical practice and is amongst the most common of the imaging predictors of OS to be investigated [6]. There are now several methods available for automated or semi-automated segmentation of GBM [7]. Tumours can be segmented into different regions, such as the enhancing and necrotic tumour, and peritumoural ‘oedema’. It has therefore become more feasible to integrate various definitions of ‘tumour volume’ into prognostic models, including in larger institutional datasets [6,8,9].

Published modelling studies have yielded inconsistent data regarding the prognostic effect of tumour diameter and volume [6,10,11,12,13]. Pre-treatment tumour size might be expected to impact on patient outcome as it could reflect the number of tumour clonogens that require ablation by conventional cytotoxic treatments [14,15]. Some of this inconsistency may be due to the sample sizes that are used to derive the prognostic models, since large cohort studies of GBM have demonstrated a weak prognostic effect of tumour diameter [13] but smaller studies have varied, with some showing positive association and some showing no relationship between diameter and prognosis [12,16,17]. Similar inconsistent findings are observed for tumour volume [6,18,19]. As well as variation in sample size (the aforementioned studies had a median sample size of 215 and range of 30–20,821), some of the inconsistency may reflect variations in handling continuous variables during statistical modelling, for example leading to dichotomisation [20], assumptions of a linear relationship to outcome [6], and use of univariable model significance to select predictors [17]. All these choices are known to impact upon the modelling process and may impact the ability to determine accurate prognostic effects of the candidate predictors [21,22].

There are a number of ways to select predictors for multivariable prognostic modelling and to evaluate the uncertainty or instability that might arise from choosing predictors in small samples or using univariable significance [21,22]. This includes the use of internal validation strategies such as data resampling (i.e., bootstrapping) to estimate uncertainty in effect size and predictor selection; however, it has been infrequently assessed in the prognostic modelling of GBM survival despite its importance [4].

Our hypothesis is that inconsistencies in the literature are secondary to varying sample size, predictor selection strategies in multivariable modelling, and consideration of data transformation. Rather than build the best prognostic model, our purpose was to investigate the prognostic effect of tumour size in a large cohort of patients diagnosed with GBM and interrogate how the choice of sample size and consideration of non-linear transformations may impact the likelihood of finding a prognostic effect using univariable and multivariable analysis and data resampling.

## 2. Materials and Methods

### 2.1. Ethical Approval

This was a retrospective study and therefore informed patient consent was not feasible. Ethical approval and institutional data access were approved via the local ethical review committee (REC ref: 19/YH/0300, IRAS project ID: 255585).

### 2.2. Patient Selection and Clinical Predictor Definitions

All consecutive patients (our institute classifies patients who are 16 years and over as adults) with histologically proven GBM according to the 2021 World Health Organisation (WHO) classification of central nervous system tumours treated at a single tertiary referral centre between 2014 and 2020 were identified retrospectively from neuro-oncology multidisciplinary team (MDT) records. The catchment area includes 3.9–4.4 million adults and over this period, 3046 new primary brain neoplasms were reviewed at MDT, with approximately 20% diagnosed with GBM or malignant glioma [23]. The inclusion criteria were MRI performed prior to any surgery, unifocal tumour (as determined by consultant neuroradiologist with >10 years experience), and all four of the following MRI sequences acquired: T1-weighted (T1W), T2-weighted (T2W), fluid-attenuated inversion recovery (FLAIR), and gadolinium-enhanced T1W (T1Gd) sequence. The exclusion criteria were an absence of pre-operative MRI, significant degradation of imaging due to artefact presence, or tumours being multifocal at presentation, documented isocitrate dehydrogenase (IDH) mutation on immunohistochemistry, or cytogenetic testing.

Demographic, clinical, and cytogenetic data were obtained from the electronic health records using in-house software. Data included patient age, sex, and type of operation. Histopathological and cytogenetic data included histology, IDH1 and 2 mutations, and O^6^-methylguanine-DNA methyltransferase (MGMT) promoter methylation. The extent of resection was estimated by the same consultant neuroradiologist using the immediate (48–72 h) post-resection MRI and grouped based upon the amount of contrast enhancing and necrotic tumour resected: (i) 100%, (ii) ≥90%, or (iii) <90%. Adjuvant treatment was categorized as the (i) full Stupp protocol–60 Gy in 30 fractions radiotherapy with concomitant and six cycles adjuvant temozolomide; (ii) partial Stupp–60 Gy in 30 fractions radiotherapy but temozolomide discontinued during either the concomitant or adjuvant treatment phase; and (iii) non-Stupp—any other treatment protocol.

### 2.3. Data Preparation

**Figure 1:**
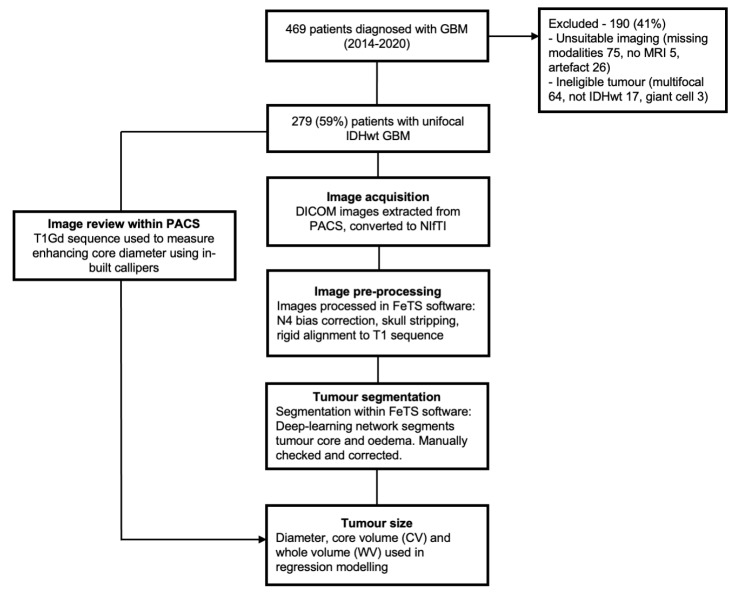
Flowchart summarising the preparation of imaging data for statistical analysis. CV—Core volume; DICOM—Digital imaging and communications in medicine; FeTS—Federated tumour segmentation software; GBM—Glioblastoma; IDHwt—Isocitrate dehydrogenase wild-type; NifTI—Neuroimaging informatics technology initiative; PACS—Picture archive and communication system; T1Gd—Gadolinium-enhanced T1-weighted imaging; WV—Whole volume.

A summary of the data preparation and numbers excluded with reasons is shown in Figure 1. Digital imaging and communications in medicine (DICOM) image preparation was performed in Python 3.9 [24]. DICOM images were retrieved from the institutional picture archive and communication system (PACS) and pseudonymised and the image acquisition parameters are summarised in Table S1. Images were converted to the Neuroimaging Informatics Technology Initiative (NIfTI) file format using the dicom2nifti (v2.3.4) package [25].

### 2.4. Image Pre-Processing and Tumour Segmentation

Semi-automated tumour segmentations were produced using the federated tumour segmentation (FeTS) software, an open-source platform available for the processing and segmentation of MRIs for patients with GBM [26]. A detailed description of the software, including packages and libraries used in FeTS, is available elsewhere [26] and utilises the same pre-processing steps used in the multimodal brain tumour image segmentation benchmark (BRATS) challenge [7] and the open-source software Cancer Imaging Phenomics Toolkit (CaPTk) [27]. The key features are outlined below.

**Figure 2:**
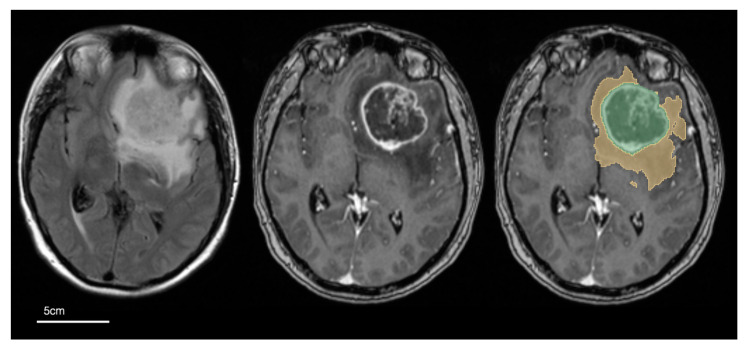
Definition of tumour volumes used in the study. Selected MRI axial images: left—fluid-attenuated inversion recovery (FLAIR); middle—gadolinium-enhanced T1-weighted (T1Gd); right—T1Gd image with overlay of core tumour segmentation (in green) and peritumoural oedema (yellow). Core volume (CV) is defined as the enhancing and necrotic component of the tumour (green) and whole volume (WV) is defined as the combination of core and peritumour oedema segmentation (green + yellow).

The T2W, T1Gd, and FLAIR sequences were rigidly co-registered first to the T1W sequence, then to the SRI24 brain atlas [28], and also spatially resampled to 1 × 1 × 1 mm voxel resolution using the Greedy registration framework [29]. Images were then skull-stripped [30] and tumour segmentation was performed with the ‘nnU-net’ deep-learning network and pre-trained model weights [31]. Tumours were automatically segmented into three volumes of interest (VOIs, mm^3^). The three VOIs were defined as (i) necrotic tumour–fluid signal intensity showing very high T2W signal and reduced T1Gd signal compared to the same area on T1W images; (ii) enhancing tumour-increased signal on T1Gd compared to the same area on T1W images and also increased T1Gd signal compared to normal white matter regions on T1Gd images; and (iii) peritumoural oedema—high FLAIR and T2W signal of the entire tumour, minus the necrotic and enhancing regions and not including ventricles or extra-axial CSF spaces [7]. Tumour masks were used to produce two tumour volumes per patient: (1) core volume (CV, cm^3^)—combination of necrotic and enhancing components and (2) whole volume (WV, cm^3^)—CV combined with the peritumoural oedema (see Figure 2).

The segmentations were checked manually and corrected using FeTS. All segmentations were checked by a board-certified neuroradiology fellow (5 years of radiology experience). Independently, 50 segmentations were also checked by a consultant neuroradiologist (>10 years of consultant neuroradiology experience) and the inter-rater concordance was compared using the dice similarity coefficient [32].

Tumour diameter was defined as the maximum axial or cranio–caudal diameter of the enhancing tumour core and was measured using the T1Gd sequence within imaging viewing software (Impax Version 6.5.3.3009, Agfa Healthcare, Mortsel, Belgium) using in-built callipers on a submillimetre scale (mm-converted to cm) by two radiology trainees (1 and 2 years radiology experience) and corrected by a board-certified neuroradiology fellow (5 years radiology experience). All manual correction and measurement were performed without knowledge of individual patient outcomes.

### 2.5. Statistical Analysis

All statistical analysis was performed in R version 4.2.2 (31 October 2022) and overseen by a career statistician (HM). Univariable association between CV, WV, or tumour diameter with overall survival (OS) was investigated using Cox regression modelling. Hazard ratios (HRs), concordance indices, and *p*-values for each model were used to assess performance. Any non-linear relationships between OS and size (volume or diameter) were explored using both logarithmic transformation and penalised spline function; the latter being implemented using a penalised spline function within the ‘survival’ package [33]. Penalised spline functions were used to assess for any trends in the data that might not be seen with a linear fit, as splines allow a smooth curve to be fit to data [34]. Overfitting to the data points is discouraged by the inclusion of a roughness penalty and the implementation does not require any pre-specification of the number of internal boundaries or knots. Model fits were assessed by plotting each tumour size parameter against the log-HR.

Multivariable association of CV, WV, or diameter to OS was also evaluated by (i) adjusting each size variable for either age, sex, type of surgery, MGMT promoter methylation status, or adjuvant oncological treatment (i.e., size variable + one clinical variable in turn) and (ii) adjusting size for all clinical parameters. As our aim was to assess the prognostic effect of tumour size, in multivariable models this was assessed using the HR for each size parameter and the Wald test *p*-value for the size variable’s coefficient rather than the overall model *p*-value.

To assess the impact that either log transformation and/or sample size could have on detecting a prognostic effect of tumour size on OS, we conducted a resampling study. Using different sample sizes (50, 100, 150, 200, 250, 258, or 279), bootstrapped samples were generated from the original dataset with replacement. For any multivariable models that were adjusted for MGMT methylation status, the maximum sample size was 258 (not 279) due to the number of cases with a known result. Bootstrapping was carried out for 1,000,000 repetitions at each sample size and for each of the tumour size variables and a Cox regression model for each tumour size variable, both with and without log transformation, was created. For univariable models, the percentage of models in which the overall model Wald test *p*-value <0.05, <0.01, and <0.001 was calculated across the 1,000,000 repetitions per sample size. For multivariable models, the percentage of models in which the Wald test *p*-value for the coefficient of tumour size (rather than the overall model Wald test significance) <0.05 was calculated across the 1,000,000 repetitions per sample size. The effect of sample size was assessed with two-sided Kolmogorov–Smirnov tests to compare the *p*-value distributions from resampling at varying sample sizes.

## 3. Results

### 3.1. Demographics of the Study Population

In total, 279 patients were included; 236 deaths occurred before the censor date of 31 October 2020. Demographic information for the GBM patients is summarised in Table 1. Overall, 39% (108/279) of patients were female and the mean age was 61 years (range 31–85 years). The mean age was 62 (range 34–85 years) for female and 61 (31–81 years) for male patients. The median OS was 12 months (95% CI 11–14 months), median follow-up time was 45 months (maximum 70 months), and 25% (71/279) patients had a surgical biopsy of their GBM. Overall, 20% (57/279) of patients had 100% resection of the tumour core and 21% (58/279) completed the full Stupp protocol of adjuvant treatment. The median (IQR) CV was 28.1 cm^3^ (12.6–50.3), WV was 103.3 cm^3^ (45.6–160.1), and tumour diameter was 4.4 cm (3.3–5.4). Histograms of tumour size (Figure S1) showed that distributions of CV and WV were slightly positively skewed and that tumour diameter was normally distributed prior to any transformation. These data confirm that our population is representative of patients diagnosed with GBM in other typical neuroscience centres [35].

### 3.2. Segmentations and Univariable Cox Models of Tumour Size

The mean (±standard deviation) dice score for core and oedema segmentations was 0.94 ± 0.05 and 0.97 ± 0.03, respectively, which are equivalent to values published in the BRATS segmentation dataset, in which multiple expert raters segment the same GBM images, and our segmentation concordance was therefore within the expected variation of inter-rater agreement [7].

Table 2 summarises the univariable Cox regression models for CV, WV, and diameter, with and without log transformation. The results of the models derived from the institutional GBM images show limited evidence for a univariable prognostic relationship between tumour volume or diameter and OS. C-indices for all models were 0.5 and all hazard ratios (HRs) crossed 1.

In Figure S2a–c, each tumour size parameter (with and without log transformation) was plotted against the log-HR. The fit of a linear function to the data was compared with the use of splines and these suggest that there was limited evidence that the tumour size parameters had a univariable prognostic relationship—the model closely followed the reference line for linear and non-linear functions. These results show that within our cohort, there was no evidence to support a univariable linear or non-linear prognostic relationship between OS and size.

In multivariable analyses, however, there was evidence of a prognostic association between size and OS when adjusting for clinical variables. A summary of the association of size variables in multivariable models with OS is shown in Table 3. CV, WV, log(WV), and diameter adjusted for type of surgery showed a statistically significant association with OS and although not significant at the 0.05 level, the HRs for log(CV) and log(diameter) were relatively wide, especially the latter, indicating uncertainty in the HR estimate. Similarly, for the model adjusted for all clinical variables, only diameter remained statistically significant at the 0.05 threshold (*p* = 0.032); however, the HR for log(CV), log(WV), and log(diameter) suggested a potential prognostic effect with relatively wider confidence intervals (and less certainty) for HR estimate of the latter two variables. The univariable and multivariable prognostic associations of each clinical variable to OS are provided in Table S2a,b. Data from our cohort of GBM patients does therefore suggest that size was associated with OS in multivariable models and whilst several related parameters did not achieve statistical significance, there was supportive evidence of a potential prognostic relationship.

### 3.3. Resampling Study

The results of the resampling experiments using univariable and multivariable models, the latter adjusted for operation type and all clinical variables, are shown in Table S3a and Table 4, respectively. In univariable models of tumour size, for all size variables, higher percentages of models with *p* < 0.05 were seen as the sample size increased and for tumour volume (CV or WV), the same was observed after log transformation, although the change was modest. For WV, 5.14 vs. 5.60% (*n* = 50 vs. *n* = 279), for CV 5.07 vs. 8.60%, and for diameter 5.43 vs. 6.39% models had *p*-values < 0.05 across all repetitions on non-transformed data. The distributions of *p*-values (across all repetitions per sample size at *n* = 50 vs. *n* = 279) differed significantly on two-sided Kolmogorov–Smirnov testing (test *p*-values < 0.0001). Table S3b,c also shows the percentages of models with *p* < 0.01 and *p* < 0.001 and this shows the same overall trend for the tumour size parameters but with successively lower percentages of models as the *p*-value threshold was lowered.

In the multivariable resampling experiment, increasing sample size increased the percentages models, either adjusted for operation type or all clinical variables (see Table S4a–d for results for multivariable models adjusted for other clinical predictors), in which the tumour size variable’s Cox regression coefficient had a Wald test *p*-value < 0.05. The impact of increasing sample size was much greater than compared with univariable modelling. Again, the distributions of *p*-values (comparing *n* = 50 vs. *n* = 279) differed significantly on two-sided Kolmogorov–Smirnov testing (all test *p*-values < 0.0001). Log transformation consistently increased the percentages of multivariable models with WV regression coefficient Wald test *p*-values < 0.05 (Table 4).

**Figure 3:**
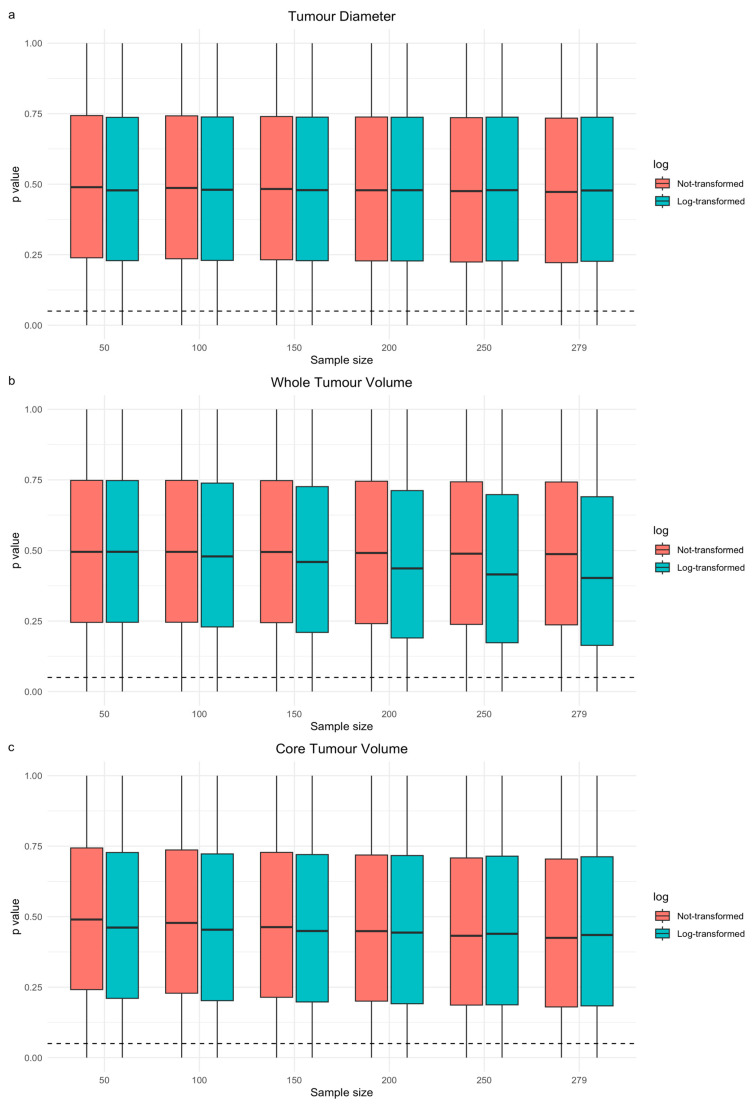
Three sets of boxplots showing the distribution of *p*-values (*y*-axis) extracted from each univariable model for tumour diameter: (**a**) whole volume (**b**) or core volume (**c**) vs. overall survival created across the 1,000,000 repetitions for each sample size (*x*-axis). Boxes outline the interquartile range of *p*-values from the resampling experiment, with median values indicated by the central thick black line. Tails represent 1.5 × the interquartile range of the distribution (outliers not shown). Models with and without log transformation are shown side by side (see figure legends). The dotted horizontal lines represent the *p*-value thresholds for statistical significance (0.05).

**Figure 4:**
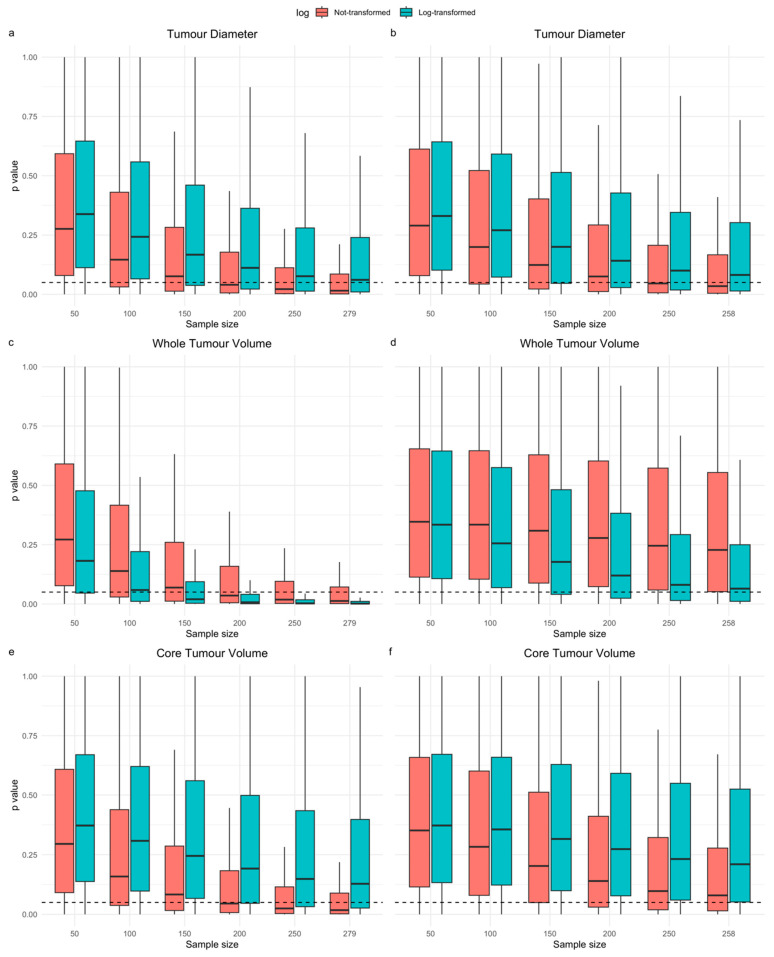
Six sets of boxplots showing the distribution of *p*-values (*y*-axis) for the Wald test of the regression coefficient of tumour diameter (**a**,**b**), whole volume (**c**,**d**), or core volume (**e**,**f**) in a multivariable Cox model vs. overall survival created across the 1,000,000 repetitions for each sample size (*x*-axis). The left column of graphs (**a**,**c**,**e**) shows results from models including the selected tumour size variable and operation type only and the right column (**b**,**d**,**f**) shows results from the multivariable model with all clinical variables and the selected tumour size variable. Boxes outline the interquartile range of *p*-values from the resampling experiment, with median values indicated by the central thick black line. Tails represent 1.5 × the interquartile range of the distribution (outliers not shown). Models with and without log transformation are shown side by side (see figure legend). The dotted horizontal lines represent the *p*-value thresholds for statistical significance (0.05).

Figure 3a–c shows the distributions of the *p*-values extracted from models during the univariable models in the resampling experiment. For CV and WV but not diameter there was modest a downward trend as the sample size increased, suggesting that this increased the probability of seeing a prognostic effect. Figure 4a–f shows the distribution of *p*-values across resamples for the regression coefficients of each tumour size variable within multivariable models, which have either been adjusted for only operation type (Figure 4a,c,e) or adjusted for all clinical variables (Figure 4b,d,f) at different sample sizes. These charts showed a much greater downward trend for all size variables and the consistent effect of log transformation in shifting the *p*-value distribution of WV downwards in multivariable modelling. Overall, results from univariable and multivariable resampling indicated that increased sample size for all size parameters and, in the case of WV, log transformation increased the chances of showing a significant univariable and multivariable association with OS.

## 4. Discussion

We set out to explore the prognostic effect of tumour volume and diameter in our institutional cohort of patients with GBM and specifically to show how the choice of sample size and consideration of non-linear transformations may impact on the chances of detecting a prognostic effect. Univariable models did not initially provide any evidence of a linear or non-linear prognostic relationship between size and OS; however, the multivariable models and resampling experiments showed that there is a prognostic role for tumour size in our dataset. Tumour diameter was prognostic in multivariable models adjusted for operation type and all clinical variables combined, whereas CV and WV were prognostic for the operation-adjusted model and showed evidence of potential prognostic effects in the combined multivariable model as well as the resampling experiments.

For WV, log transformation could also increase the probability of detecting a statistically significant effect, potentially due to the positively skewed distribution. WV might play a role in prognostication even when adjusted for the extent of tumour core resection as illustrated in our multivariable models and resampling experiments and this could be due to WV encompassing more of the infiltrated brain tissue. However, WV is infrequently explored as a candidate prognostic variable in patients with GBM [6,36,37,38]. In 65 patients, Iliadis et al. found no significant association between WV and OS using univariable Cox modelling [37] and it is unclear if any log or other transformation was considered. Palpan Flores et al. investigated the equivalent of WV in 44 IDH-wildtype GBM patients and found a significant effect of WV > 60 cm^3^ in univariable and multivariable models (adjusted HR 3.93 95% CI 1.23–10.2, *p* = 0.018) [38]. Other groups have investigated peritumoural oedema alone, rather than WV, and these studies have shown mixed results [20,36,39,40,41]. Fuster-Garcia et al. found no prognostic effect for peritumoural oedema volume in 84 patients [39], whereas Wangaryattawanich et al. showed a statistically significant effect for peritumoural oedema when dichotomising volume using a threshold of 85,000 mm^3^ in a cohort of 94 patients [20]. Although the multivariable model adjusted for all clinical parameters in the complete cohort did not show a statistically significant result for log-transformed WV, the confidence interval for its hazard ratio was relatively wide, suggesting a higher degree of uncertainty in the result. Second, the results of the multivariable resampling for the log(WV) full clinical model suggested that at the sample sizes used in the above-cited literature, there is a lower chance of detecting the potentially prognostic role than in our cohort study.

For tumour diameter and CV, which are more commonly investigated [4,6], there are several studies in similarly sized institutional datasets that did not show a prognostic effect for either CV [12,36,42] or diameter [17,43]. However, a small positive effect size has been shown in studies with larger datasets [13,18]. These findings support the initial multivariable model and experiment resampling findings, which indicate that diameter does have a small multivariable prognostic effect and that CV could potentially have prognostic effects, after adjustment for other clinical parameters and in larger samples. Li et al. for example found that contrast enhancing the tumour volume had a small but statistically significant effect in a cohort of 1226 GBM patients (HR 1.004 95% CI 1.002–1.006, *p* < 0.001) [18]. Senders et al. also showed a small (relative survival rate for a 10 cm increase in diameter-0.99 95% CI 0.99–1.00) but significant effect of tumour diameter in 16,656 patients [13]. Whilst it could be argued that such a small effect size is not clinically significant, the aim of our study was not to produce a prognostic model for clinical use but to identify the barriers to detecting potentially significant effects in GBM prognostic models and suggest that resampling and data transformation can have a role in highlighting the uncertainty of predictor selection in relatively small datasets. Future studies would benefit from leveraging multi-institutional networks [26] or online imaging repositories to further increase statistical power for studying clinically relevant size parameters including volumetric assessments.

An important consideration in regression modelling is the inclusion of any non-linear transformation of variables [44], although this is not routinely documented in prognostic modelling studies in GBM [4,6]. The advantages include more flexible modelling of continuous variables that might not have a simple linear relationship to the outcome but this comes with the drawback of potentially overfitting a model to the development dataset. In our univariable resampling study, logarithmic transformation led to a modestly higher rate of significant models for WV and this effect was much greater in multivariable models. The results of our resampling study point to a possible explanation as to why some prognostic models that assume linear relationships between volume and outcome return non-significant results, particularly in the case of WV. In the present study, the WV shows a small positive skew and large range that might explain why log transformation increased the chances of detecting a potential prognostic relationship for WV.

Bootstrapping (resampling with replacement) as a method for exploring model uncertainty has been described elsewhere in the statistics literature [21,22,44] and was used in our resampling study to demonstrate the variability in the prognostic effect of tumour size. By resampling a dataset multiple times, researchers can identify the uncertainty in multiple aspects of the model-building process, such as feature selection, internal validation of model accuracy, and model stability [21]. Our study suggests that when selecting one of these size variables in GBM prognostic models based on univariable model significance, there could be up to 5–10% uncertainty in whether they might be statistically significant and therefore included in a multivariable model if using this as a selection criterion. The uncertainty is shown to be even greater in the multivariable resampling and there could be a large amount of uncertainty as to whether a variable is prognostic based on a limited sample size. This is one of the reasons that this approach of univariable screening of candidate predictors is generally not recommended for multivariable model building and also why focusing on *p*-values in multivariable modelling may lose some of the important information in estimating prognostic effects [21,22].

There are several limitations of our study. The MRI acquisition parameters were heterogeneous, especially slice thickness, and this could have impacted upon the accuracy of volume measurements. However, the spatial resampling of images to an isotropic 1 mm^3^ voxel resolution should have reduced the impact of acquisition heterogeneity. Furthermore, the dataset represents a retrospective real-world clinical dataset, which in our institution’s routine practice is likely to include different imaging acquisitions due to patients being referred from other centres, with their own (varying) MRI protocols. A proportion of our patients had to be excluded due to a lack of the necessary MRI sequences for the deep-learning segmentation algorithm. The efficiency of a semi-automated segmentation approach outweighed the potential limitation of a reduced sample size. We investigated only three size variables but there are many others described in the literature. Whilst this could be deemed a limitation of our approach, we did not aim to provide a comprehensive study of the prognostic role of all possible tumour size parameters in GBM but to investigate some of the methodological issues affecting this question that could be applied to any of the other continuous measures of tumour size in GBM.

## 5. Conclusions

In summary, univariable models derived from our large well-curated institutional dataset of patients with GBM showed limited evidence to support a linear or non-linear prognostic association between size and patient outcome; however, the multivariable models did support a prognostic role for tumour size. The diameter showed a significant multivariable association with survival, whereas CV and log(WV) showed significant effects when adjusted for operation type and potential for an effect in the full clinical model. Importantly, resampling demonstrated the impact that increasing sample size and log transformation (for WV) had in increasing the ability to detect prognostic relationships in univariable and multivariable models.

## Figures and Tables

**Table 1:** Summary of patient demographics and treatment (*n* = 279).

Demographic	Value
Age, years–median (IQR)	62 (55–68)
Gender–no. female (%)	108 (39%)
Surgical treatment–no. (%)	
Biopsy	71 (25%)
100% resected ^a^	57 (20%)
≥90% resected ^a^	86 (31%)
<90% resected ^a^	65 (23%)
Adjuvant oncology treatment–no. (%)	
No Stupp	150 (54%)
Full Stupp ^b^	58 (21%)
Partial Stupp ^c^	71 (25%)
MGMT methylation–no. (% of known) ^d^	103 (40%)
Overall survival, months–median (95% CI)	12 (11–14)
Maximum tumour diameter, cm–median (IQR)	4.4 (3.3–5.4)
Core volume, cm^3^–median (IQR)	28.1 (12.6–50.3)
Whole volume, cm^3^–median (IQR)	103 (45.6–160)

^a^ Percentage of contrast enhancing and necrotic tumour core removed; ^b^ Completed 60Gy in 30 fractions radiotherapy with concomitant temozolomide and six cycles adjuvant temozolomide; ^c^ Completed 60 Gy in 30 fractions radiotherapy with concomitant temozolomide and began adjuvant temozolomide; ^d^ 258 cases with the result known; IQR—Interquartile range; MGMT—O^6^-methylguanine-DNA methyltransferase; CI—Confidence interval.

**Table 2:** Summary of univariable Cox proportional hazards models for each tumour size parameter (whole volume, core volume, and diameter) for predicting overall survival (*n* = 279).

	Whole Volume (WV)		Core Volume (CV)		Tumour Diameter	
	WV	log(WV)	CV	log(CV)	Diameter	log(Diameter)
C (95% CI)	0.5 (0.46–0.54)	0.5 (0.46–0.54)	0.5 (0.46–0.54)	0.5 (0.46–0.54)	0.5 (0.46–0.54)	0.5 (0.46–0.54)
HR (95% CI)	1 (1–1)	1.1 (0.81–1.6)	1 (1–1)	0.95 (0.71–1.3)	1 (0.93–1.1)	0.94 (0.43–2)
*p* value	0.784	0.475	0.539	0.704	0.745	0.875

C-index—Concordance index; HR—Hazard ratio; WV—Whole volume; CV—Core volume.

**Table 3:** Table of the prognostic effect of each tumour size parameter (whole volume, core volume, and diameter) within multivariable models predicting overall survival that have been adjusted for selected clinical variables (*n* = 279).

	Tumour Diameter				Whole Volume (WV)				Core Volume (CV)			
	Diameter		log(Diameter)		WV		log(WV)		CV		log(CV)	
Variable	HR (95% CI)	*p*	HR (95% CI)	*p*	HR (95% CI)	*p*	HR (95% CI)	*p*	HR (95% CI)	*p*	HR (95% CI)	*p*
Age	1.01 (0.92–1.10)	0.91	0.86 (0.39–1.88)	0.70	1.00 (1.00–1.00)	0.90	1.09 (0.78–1.5)	0.61	1.00 (1.00–1.01)	0.56	0.93 (0.7–1.23)	0.6
Gender	1.00 (0.92–1.10)	0.93	0.87 (0.40–1.89)	0.73	1.00 (1.00–1.00)	0.99	1.09 (0.78–1.5)	0.61	1.00 (1.00–1.01)	0.71	0.91 (0.69–1.22)	0.54
Type of surgery	1.14 (1.02–1.26)	0.016	2.4 (0.96–5.98)	0.063	1.00 (1.00–1.00)	0.013	1.90 (1.28–2.82)	0.001	1.01 (1.00–1.01)	0.018	1.29 (0.93–1.79)	0.13
Adjuvant oncology treatment	1.00 (0.91–1.09)	0.93	0.82 (0.37–1.79)	0.61	1.00 (1.00–1.00)	0.99	1.05 (0.75–1.47)	0.76	1.00 (1.00–1.01)	0.67	0.92 (0.69–1.23)	0.58
MGMT methylation	1.02 (0.93–1.12)	0.70	0.96 (0.43–2.18)	0.93	1.00 (1.00–1.00)	0.98	1.10 (0.78–1.5)	0.60	1.00 (1.00–1.01)	0.71	0.94 (0.70–1.26)	0.68
Age + Gender + Surgery + Oncology + MGMTa	1.12 (1.01–1.25)	0.032	2.3 (0.91–6.01)	0.076	1.00 (1.00–1.00)	0.24	1.45 (0.98–2.14)	0.06	1.00 (1.00–1.01)	0.072	1.24 (0.89–1.7)	0.20

Each row of the table presents the results from the Cox proportional hazards models that include the tumour size variable specified by the column name (either diameter, core, or whole volume or their log-transformed versions) and the clinical variable indicated in the ‘Variable’ column. The stated hazard ratios (and 95% confidence intervals) refer to the selected tumour size variable and not the clinical variable indicated in the ‘Variable’ column. The stated *p*-values refer to the Wald test for the regression coefficient of the tumour size variable and not the overall multivariable Cox model significance/*p*-value. HR—Hazard ratio; CI—Confidence interval; CV—Core volume; WV—Whole volume; MGMT—O^6^-methylguanine-DNA methyltransferase; an = 258, cases with known MGMT result.

**Table 4:** Percentage of multivariable models, either adjusted for operation only (left) or adjusted for all clinical variables (right), in which the tumour size variable’s regression coefficient has a Wald-test *p*-value < 0.05 during the resampling study.

Adjusted for Operation Type							Adjusted for Age + Gender + Surgery + Oncology + MGMT						
Sample Size	Tumour Diameter		Whole Volume (WV)		Core Volume (CV)		Sample Size	Tumour Diameter		Whole Volume (WV)		Core Volume (CV)	
	Diameter	log(Diameter)	WV	log(WV)	CV	log(CV)		Diameter	log(Diameter)	WV	log(WV)	CV	log(CV)
50	19.01	14.78	19.45	26.30	17.15	11.93	50	19.53	16.39	14.95	15.54	14.87	12.93
100	31.24	21.54	32.15	47.22	28.95	16.28	100	26.74	20.39	16.13	20.97	19.15	13.78
150	42.94	28.75	44.56	64.84	40.92	21.16	150	35.14	26.01	18.29	27.80	25.15	16.32
200	53.50	36.03	55.58	77.80	51.63	26.03	200	43.47	32.24	20.47	34.84	31.77	19.38
250	62.47	42.82	65.10	86.42	61.30	30.92	250	51.34	38.30	23.06	41.89	38.19	22.67
279	67.16	46.66	69.87	89.94	66.05	33.61	258 ^a^	55.67	41.79	24.57	45.72	41.89	24.50

The percentages in the table cells represent the percentage of resamples in which the Wald test *p*-value for the regression coefficient of the selected tumour size variable (each column) was <0.05. The left side of the table shows the results when each tumour size variable was adjusted only for the type of operation (i.e., size + operation entered into the Cox model) and the right side of the table shows the results when size was adjusted for all clinical variables stated. CV—Core volume; WV—Whole volume; MGMT—O^6^-methylguanine-DNA methyltransferase; ^a^ Maximum sample size limited to 258 due to the number of cases with a known MGMT result.

## Data Availability

The data presented in this study have restrictions due to patient confidentiality and are therefore not publicly available; however, they can be made available on reasonable request from the corresponding author.

## Supplementary Materials

The following supporting information can be downloaded at https://www.mdpi.com/article/10.3390/cancers16071301/s1. Figure S1: Histograms of tumour diameter, core tumour, and whole tumour volumes before and after logarithmic transformation; Figure S2a–c: Non-linear modelling of (a) tumour diameter, (b) whole tumour volume, and (c) core tumour volume with log transformation and penalised splines; Table S1: Summary of MRI acquisition parameter per imaging sequence; Table S2a,b: (a) Univariable and (b) multivariable association between clinical variables and overall survival; Table S3a–c: Percentage of univariable tumour size models with model (a) *p*-values < 0.05, (b) *p*-values < 0.01, and (c) *p*-values < 0.001 during the resampling study; Table S4a–d: Percentage of resamples in which the multivariable tumour size model (a) adjusted for patient age (i.e., age + tumour size in model), (b) adjusted for patient gender (i.e., gender + tumour size in model), (c) adjusted for adjuvant oncology treatment received (i.e., oncology treatment + tumour size in model), and (d) adjusted for MGMT promoter methylation (i.e., MGMT methylation + tumour size in model) has a tumour size regression coefficient Wald test *p*-value < 0.05.
