# Supplementary material for: Tumour Size and Overall Survival in a Cohort of Patients with Unifocal Glioblastoma: A Uni- and Multivariable Prognostic Modelling and Resampling Study
Source: Cancers (Basel). 2024 Mar 27;16(7):1301. doi: 10.3390/cancers16071301 (PMC11011077; doi:10.3390/cancers16071301)
Supplement: Supplementary file 1 [file cancers-16-01301-s001.zip › cancers-2896786-supplementary.pdf]

Figure S1. Histograms of tumour diameter, core tumour, and whole tumour volumes before and after logarithmic transformation

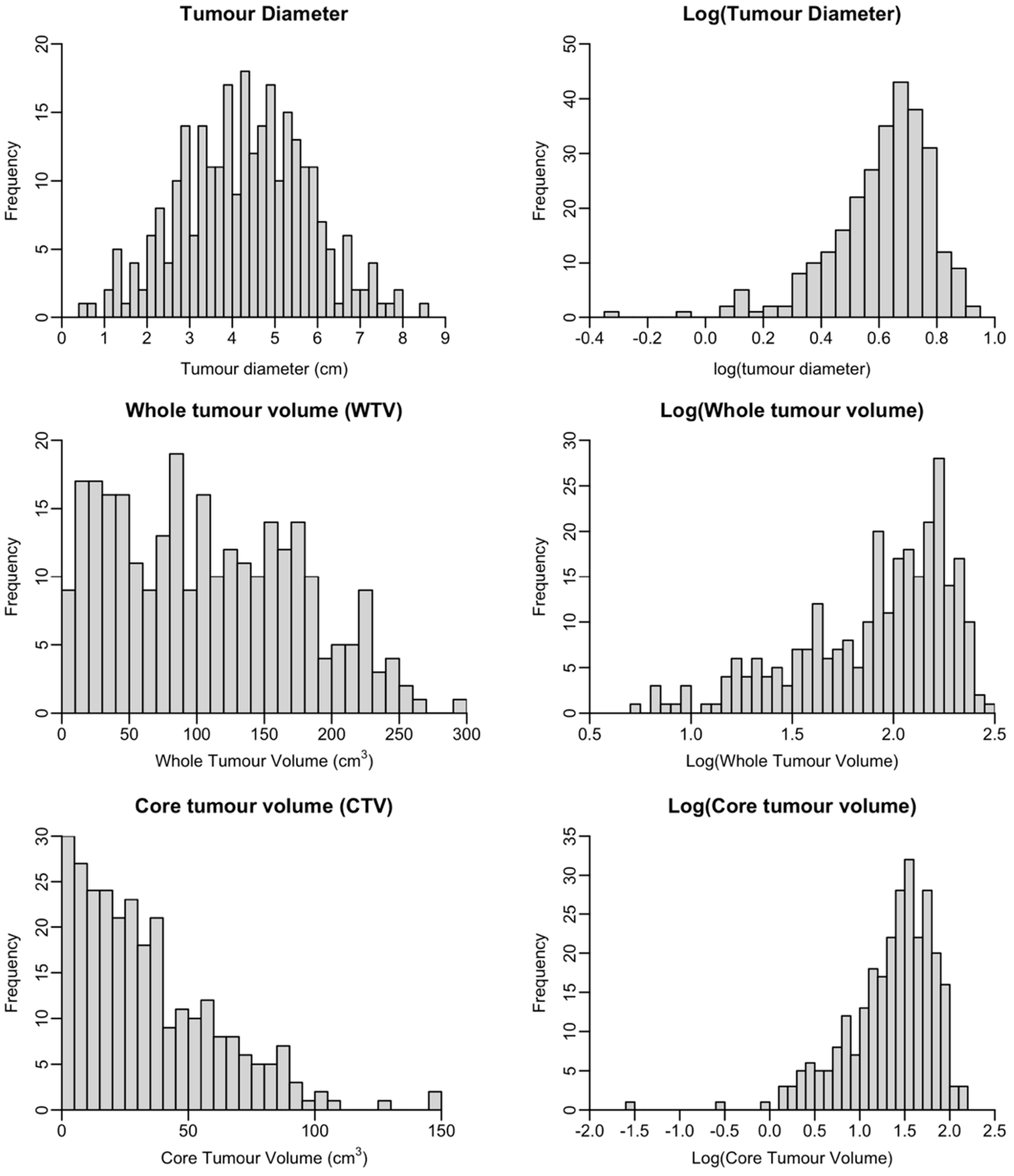

Figure S2a. Non-linear modelling of tumour diameter with log-transformation and penalised splines.

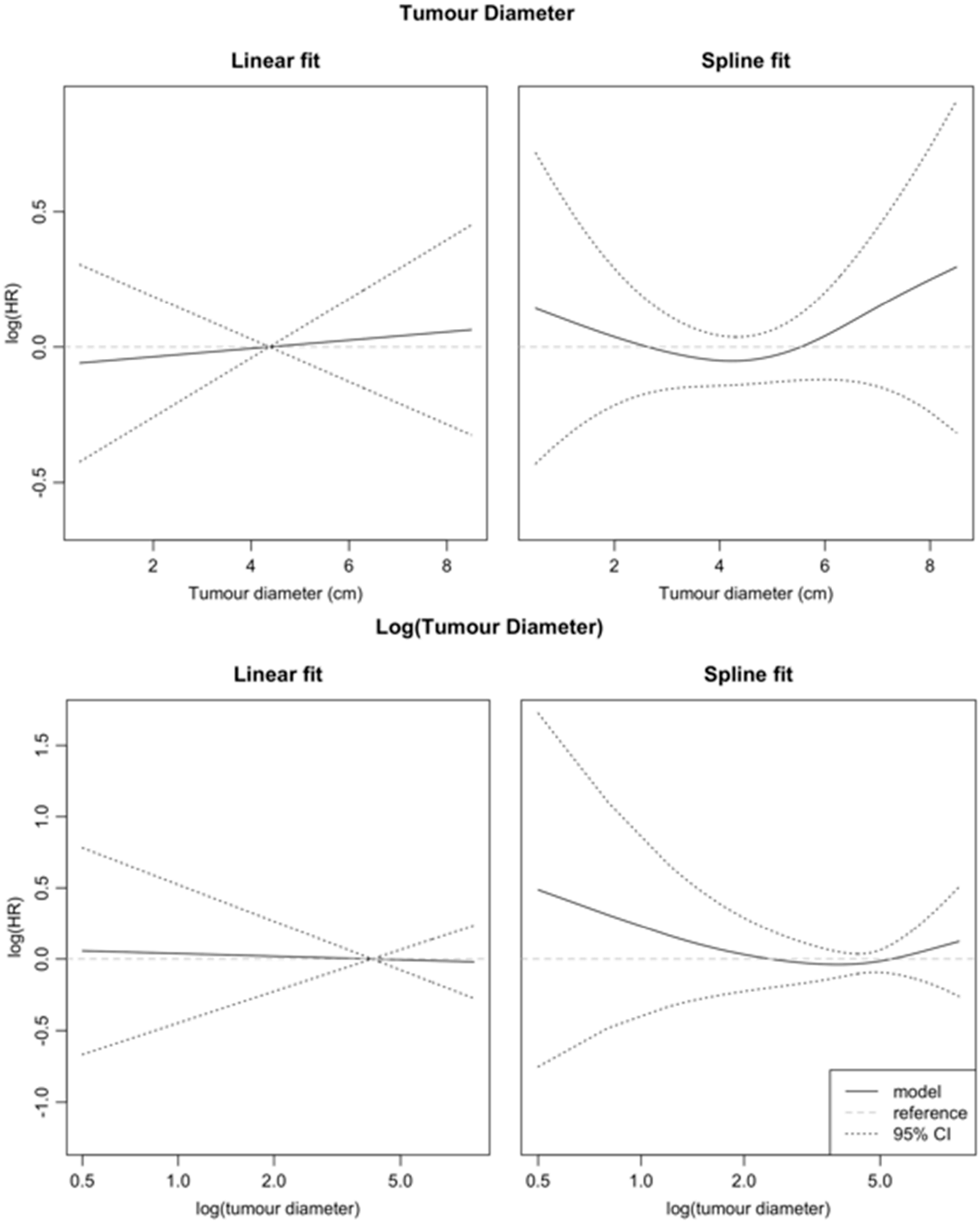

Figure S2b. Non-linear modelling of whole tumour volume with log-transformation and penalised splines.

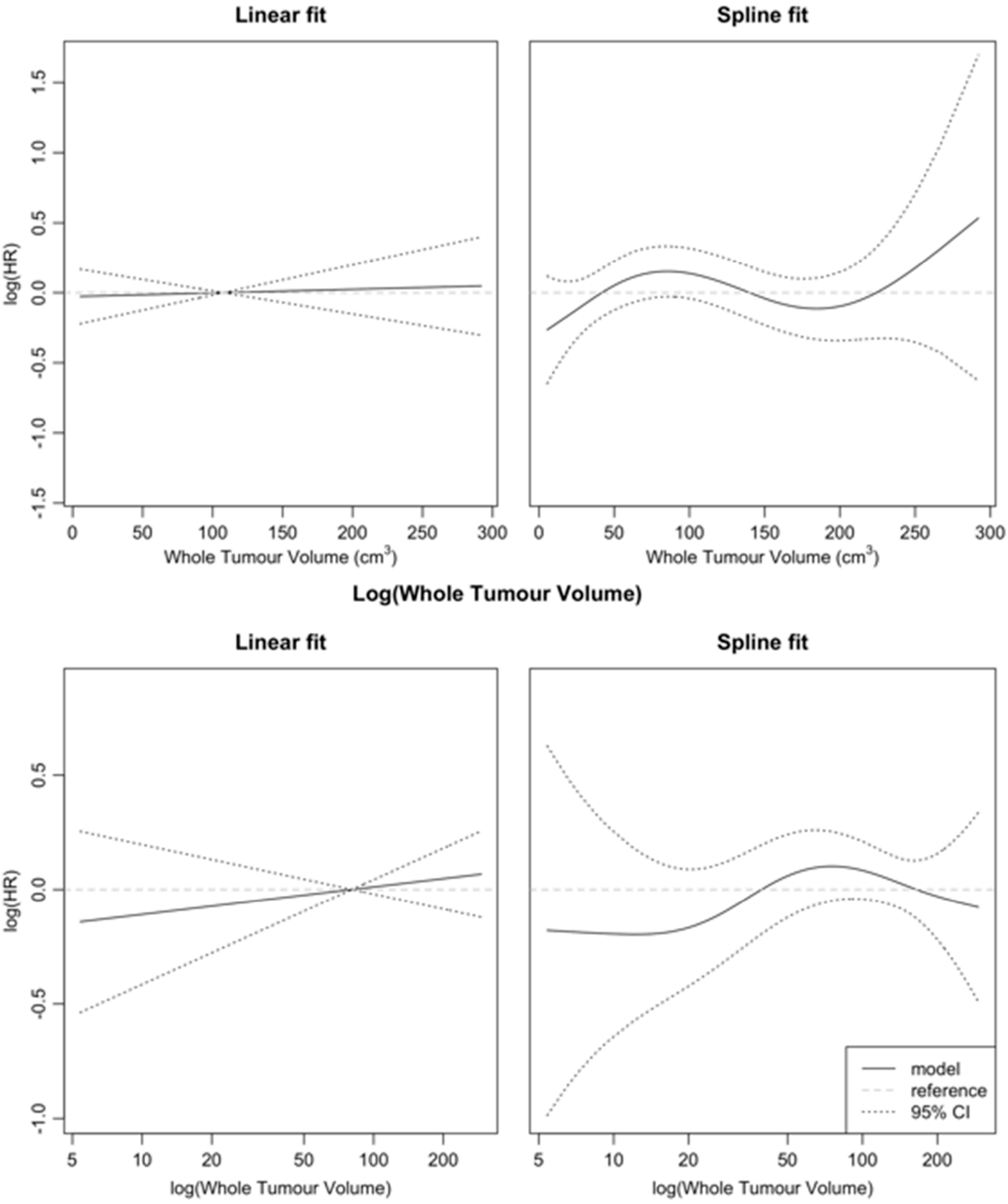

Figure S2c. Non-linear modelling of core tumour volume with log-transformation and penalised splines

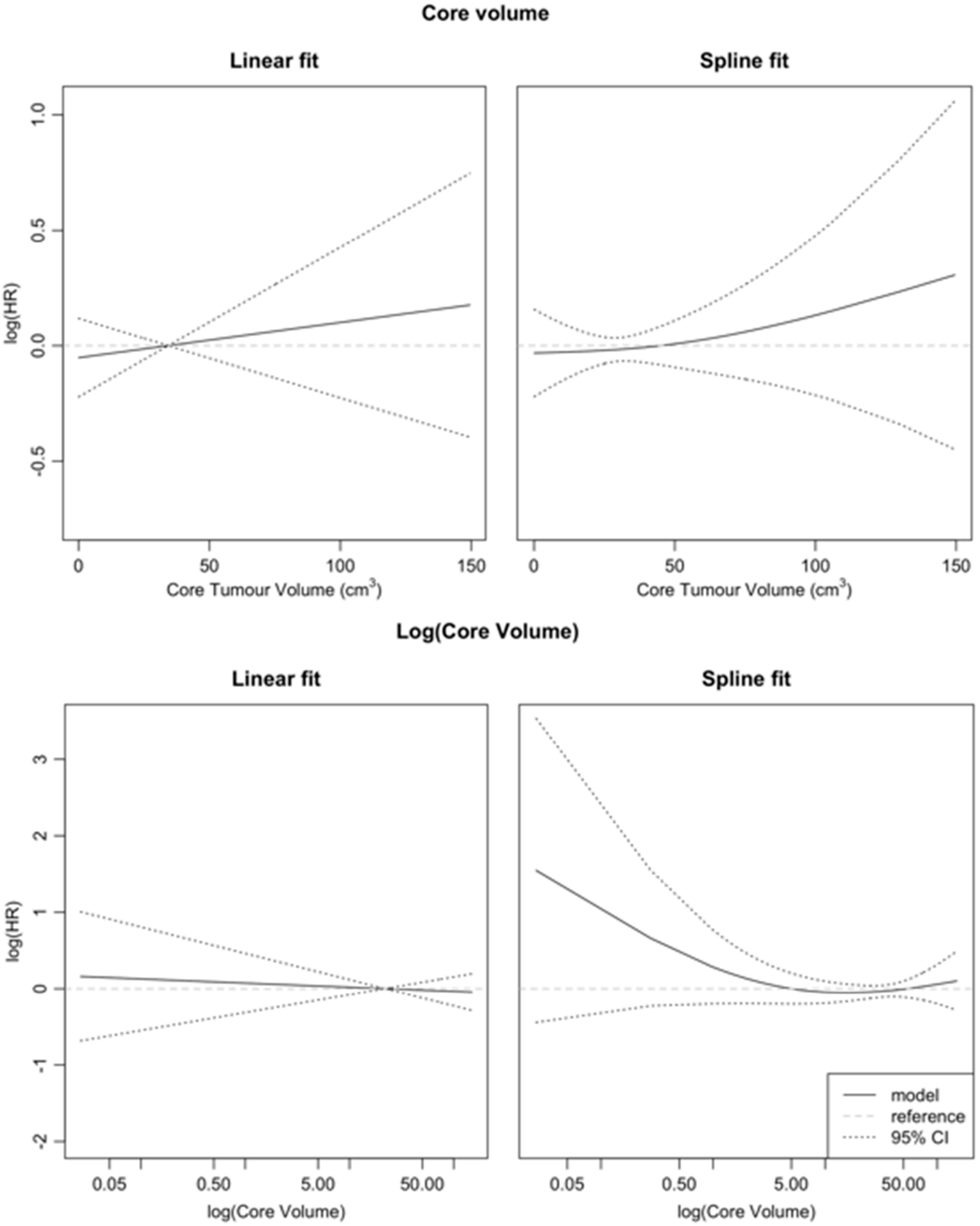

Supplementary Figure 2a-c. For each 2 x 2 plot, the top row shows models before log-transformation and the bottom row shows them after log-transformation. The graph on the left illustrates a linear fit to the data points and the graph on the right shows a fit of a penalised spline function to fit the data points more closely. HR—hazard ratio.

Table S1. Summary of MRI acquisition parameter per imaging sequence

Sequence	Parameter summary
T1	Slice thickness 5mm (0 – 5 mm), spacing between slices 5.5 mm (0.6 – 7mm), echo time 7.8 ms (3.0 – 58 ms), repetition time 550 ms (7.0 – 3200 ms), field strength 1.5 T (1.5 – 3.0 T), flip angle 90 deg (8 – 150 deg)
T2	Slice thickness 5 mm (1.2 – 7 mm), spacing between slices 5.5 mm (0.6 – 7.7 mm), echo time 98 ms (25 – 171 ms), repetition time 5268.6 ms (660 – 6600 ms), field strength 1.5 T (1.5 – 3.0 T), flip angle 150 deg (20 – 180 deg)
FLAIR	Slice thickness 5mm (0.7 – 5 mm), spacing between slices 5.5mm (0.6 – 7 mm), echo time 109 ms (82 – 474 ms), inversion time 2500 ms (1660 – 2880 ms), repetition time 9000 ms (4610 – 14788 ms), field strength 1.5 T (1.5 – 3.0 T), flip angle 150 deg (90 – 180 deg)
T1Gd	Slice thickness 1.1 mm (0 – 7 mm), spacing between slices 5.9 mm (0.5 – 7.7 mm), echo time 3.9 ms (2.3 – 46 ms), repetition time 700 ms (7.5 – 3200 ms), field strength 1.5 T (1.5 – 3.0 T), flip angle 15 deg (8 – 150 deg)

Values for acquisition parameters are presented as median (range). FLAIR—Fluid attenuated inversion recovery. T1Gd—Post-gadolinium T1-weighted imaging.

Table S2a. Univariable association between clinical variables and overall survival.

Characteristic	N	Event N	HR	95% CI	p-value
Age	279	236	1.01	1.00, 1.03	0.060
Sex	279	236			
Female			—	—	
Male			1.26	0.96, 1.64	0.091
Operation	279	236			
Biopsy			—	—	
100% resected[a]			0.34	0.23, 0.50	<0.001
≥90% resected[a]			0.38	0.27, 0.54	<0.001
<90% resected[a]			0.50	0.35, 0.71	<0.001
Stupp	279	236			
No Stupp			—	—	
Full Stupp[b]			0.29	0.20, 0.41	<0.001
Partial Stupp[c]			0.49	0.36, 0.67	<0.001
MGMT	258[d]	219			
Unmethylated			—	—	
Methylated			0.56	0.42, 0.74	<0.001

HR—Hazard ratio; CI—Confidence interval.

[a]Percentage of contrast enhancing and necrotic tumour core removed.

[b]Completed 60Gy in 30 fractions radiotherapy with concomitant temozolomide and 6 cycles adjuvant temozolomide.

[c]Completed 60Gy in 30 fractions radiotherapy but stopped temozolomide either during radiotherapy or adjuvant course.

[d]Number of cases with known result.

Table S2b. Prognostic effect of each clinical variable within a multivariable model adjusted for all other clinical variables.

Characteristic	HR	95% CI	p-value
Age	1.00	0.98, 1.02	0.98
Sex			
Female	—	—	
Male	1.31	0.99, 1.75	0.061
Operation			
Biopsy	—	—	
100% resected[a]	0.38	0.25, 0.56	<0.001
≥90% resected[a]	0.36	0.25, 0.52	<0.001
<90% resected[a]	0.43	0.29, 0.63	<0.001
Stupp			
No Stupp	—	—	
Full Stupp[b]	0.34	0.23, 0.50	<0.001
Partial Stupp[c]	0.56	0.39, 0.79	<0.001
MGMT			
Unmethylated	—	—	
Methylated	0.67	0.50, 0.90	0.007

HR—Hazard ratio, CI—Confidence interval. Multivariable model included age, sex, operation type, Stupp status, and MGMT methylation.

n = 258 (219 events) = cases with complete results for all clinical variables.

[a]Percentage of contrast enhancing and necrotic tumour core removed.

[b]Completed 60Gy in 30 fractions radiotherapy with concomitant temozolomide and 6 cycles adjuvant temozolomide.

[c]Completed 60Gy in 30 fractions radiotherapy with concomitant temozolomide and began adjuvant temozolomide.

Table S3a. Percentage of univariable tumour size models with model p-values <0.05 during the resampling study.

Sample size	Tumour Diameter		Whole Volume (WV)		Core Volume (CV)	
	Diameter	log(diameter)	WV	log(WV)	CV	log(CV)
50	5.43	5.95	5.14	5.20	5.07	7.07
100	5.60	5.94	5.12	5.94	5.73	7.57
150	5.84	6.00	5.21	6.93	6.53	8.00
200	6.05	6.04	5.35	8.00	7.32	8.36
250	6.25	6.07	5.53	9.04	8.15	8.68
279	6.39	6.13	5.60	9.70	8.60	8.94

CV— Core volume; WV— Whole volume.

Table S3b. Percentage of univariable tumour size models with model p-values <0.01 during the resampling study.

Sample size	Tumour Diameter		Whole Volume (WV)		Core Volume (CV)	
	Diameter	log(diameter)	WV	log(WV)	CV	log(CV)
50	1.12	1.31	1.05	1.06	0.91	1.66
100	1.16	1.26	1.03	1.26	1.13	1.91
150	1.26	1.31	1.05	1.57	1.39	2.12
200	1.34	1.33	1.09	1.90	1.66	2.26
250	1.40	1.34	1.15	2.29	1.93	2.39
279	1.44	1.33	1.16	2.51	2.08	2.49

CV— Core volume; WV— Whole volume.

Table S3c. Percentage of univariable tumour size models with model p-values <0.001 during the resampling study.

Sample size	Tumour Diameter		Whole volume (WV)		Core Volume (CV)	
	Diameter	log(diameter)	WV	log(WV)	CV	log(CV)
50	0.11	0.15	0.11	0.11	0.08	0.21
100	0.12	0.14	0.10	0.14	0.10	0.27
150	0.14	0.14	0.10	0.18	0.14	0.30
200	0.15	0.15	0.11	0.22	0.18	0.34
250	0.16	0.15	0.11	0.30	0.22	0.36
279	0.17	0.15	0.12	0.33	0.24	0.39

CV— Core volume; WV—Whole volume.

Table S4a. Percentage of resamples in which the multivariable tumour size model adjusted for patient age (i.e., age + tumour size in the model) has a tumour size regression coefficient Wald test p-value <0.05.

Sample size	Tumour Diameter		Whole Volume (WV)		Core Volume (CV)	
	Diameter	log(diameter)	WV	log(WV)	CV	log(CV)
50	5.61	6.61	5.60	5.00	5.71	7.70
100	5.53	6.75	5.30	5.00	6.30	8.30
150	5.47	6.96	5.22	5.42	7.01	8.87
200	5.44	7.23	5.21	5.90	7.68	9.37
250	5.43	7.49	5.19	6.39	8.43	9.98
279	5.45	7.64	5.25	6.65	8.82	10.28

CV—Core volume; WV—Whole volume.

Table S4b. Percentage of resamples in which the multivariable tumour size model adjusted for patient gender (i.e., gender + tumour size in the model) has a tumour size regression coefficient Wald test p-value <0.05.

Sample size	Tumour Diameter		Whole Volume (WV)		Core Volume (CV)	
	Diameter	log(diameter)	WV	log(WV)	CV	log(CV)
50	5.40	6.63	5.58	4.74	5.33	8.30
100	5.30	6.65	5.28	4.78	5.54	9.08
150	5.27	6.78	5.19	5.10	5.77	9.82
200	5.24	6.95	5.12	5.57	6.07	10.62
250	5.27	7.15	5.07	6.03	6.32	11.39
279	5.20	7.28	5.10	6.33	6.48	11.90

CV—Core volume; WV—Whole volume.

Table S4c. Percentage of resamples in which the multivariable tumour size model adjusted for adjuvant oncology treatment received (i.e., oncology treatment + tumour size in the model) has a tumour size regression coefficient Wald test p-value <0.05.

Sample size	Tumour Diameter		Whole Volume (WV)		Core Volume (CV)	
	Diameter	log(diameter)	WV	log(WV)	CV	log(CV)
50	9.50	9.74	7.59	6.94	7.41	10.43
100	9.30	10.10	6.97	6.65	7.68	11.08
150	9.18	10.48	6.72	6.65	7.92	11.73
200	9.08	10.96	6.51	6.67	8.23	12.30
250	9.08	11.42	6.50	6.83	8.53	12.94
279	9.03	11.77	6.42	6.94	8.77	13.22

CV—Core volume; WV—Whole volume.

Table S4d. Percentage of resamples in which the multivariable tumour size model adjusted for MGMT promoter methylation (ie. MGMT methylation + tumour size in model) has a tumour size regression coefficient Wald test p-value <0.05.

Sample size	Tumour Diameter		Whole Volume (WV)		Core Volume (CV)	
	Diameter	log(diameter)	WV	log(WV)	CV	log(CV)
50	6.52	8.29	6.31	5.85	5.89	10.80
100	6.78	8.51	6.03	5.86	6.07	11.44
150	7.05	8.55	5.89	6.26	6.27	11.87
200	7.29	8.57	5.79	6.75	6.53	12.23
250	7.63	8.54	5.75	7.34	6.86	12.58
258[a]	7.80	8.52	5.74	7.63	6.97	12.74

CV—Core volume; WV—Whole volume.

[a]Maximum sample size limited to 258 due to the number of cases with a known MGMT result.
